# MicroRNA profiling study reveals miR-150 in association with metastasis in nasopharyngeal carcinoma

**DOI:** 10.1038/s41598-017-10695-2

**Published:** 2017-09-20

**Authors:** Patrick Ying-Kit Yue, Wai-Yan Ha, Chi-Chiu Lau, Florence Man-Fung Cheung, Anne Wing-Mui Lee, Wai-Tong Ng, Roger Kai-Cheong Ngan, Chun-Chung Yau, Dora Lai-Wan Kwong, Hong-Lok Lung, Nai-Ki Mak, Maria Li Lung, Ricky Ngok-Shun Wong

**Affiliations:** 1 Department of Biology, Faculty of Science, Hong Kong Baptist University, Hong Kong, China; 2 Center for Nasopharyngeal Carcinoma Research, University of Hong Kong, Hong Kong, China; 3 Department of Clinical Pathology, Pamela Youde Nethersole Eastern Hospital, Hong Kong, China; 4 Department of Clinical Oncology, Pamela Youde Nethersole Eastern Hospital, Hong Kong, China; 5 Department of Clinical Oncology, Queen Elizabeth Hospital, Hong Kong, SAR People’s Republic of China; 6 Department of Oncology, Princess Margaret Hospital, Hong Kong, SAR People’s Republic of China; 7 Department of Clinical Oncology and Center for Cancer Research, University of Hong Kong, Hong Kong, SAR People’s Republic of China; 8 Department of Clinical Oncology, University of Hong Kong, Hong Kong, China

## Abstract

MicroRNAs (miRNAs) are small non-coding RNAs that play a crucial role in pathogenesis of human cancers. Several miRNAs have been shown to involve in nasopharyngeal carcinoma (NPC) pathogenesis through alteration of gene networks. A global view of the miRNA expression profile of clinical specimens would be the best way to screen out the possible miRNA candidates that may be involved in disease pathogenesis. In this study, we investigated the expression profiles of miRNA in formalin-fixed paraffin-embedded tissues from patients with undifferentiated NPC versus non-NPC controls using a miRNA real-time PCR platform, which covered a total of 95 cancer-related miRNAs. Hierarchical cluster analysis revealed that NPC and non-NPC controls were clearly segregated. Promisingly, 10 miRNA candidates were differentially expressed. Among them, 9 miRNAs were significantly up-regulated of which miR-205 and miR-196a showed the most up-regulated in NPC with the highest incidence percentage of 94.1% and 88.2%, respectively, while the unique down-regulated miR-150 was further validated in patient sera. Finally, the *in vitro* gain-of-function and loss-of-function assays revealed that miR-150 can modulate the epithelial-mesenchymal-transition property in NPC/HK-1 cells and led to the cell motility and invasion. miR-150 may be a potential biomarker for NPC and plays a critical role in NPC tumourigenesis.

## Introduction

Nasopharyngeal carcinoma (NPC) is a squamous cell carcinoma that usually develops around the ostium of the Eustachian tube in the lateral wall of the nasopharynx^1^. NPC can be divided into two categories, keratinising squamous-cell carcinoma and non-keratinising carcinoma (WHO Classification 1991)^2,3^. The former shows squamous differentiation with the presence of intercellular bridges and/or keratinisation over most of its extent. The later comprises a differentiated type of non-keratinising carcinoma and an undifferentiated type. This type of tumour is generally more radiosensitive and has stronger relationships with the Epstein-Barr virus (EBV). NPC is rare in most populations worldwide but it is frequently seen among ethnic Chinese in the Southern China (in particular, Hong Kong and the province of Guangdong) or Southeast Asia^4^. According to the report by the Hong Kong Cancer Registry (2011), NPC is the seventh most common malignant tumour in the middle-aged patients (45–64 years) with the highest mortality rate in Hong Kong. It accounted for 3.8% of all cancer new cases^5^. The clinical symptoms of NPC are non-specific, which may include a lump in the neck region, blood-stained post nasal drip, tinnitus, hearing impairment, and headache: early disease can be asymptomatic, early detection is often difficult.

MicroRNAs (miRNAs) are short non-coding RNA molecules (about 20–23 bp) for gene regulation at the post-transcriptional level by inhibition of translation or by degradation of the target mRNA. Increasing evidence showed that miRNA can function as tumour suppressors or oncogenes which play a major role in the pathogenesis of the tumour^6^. MiRNAs are involved in all the molecular and biological processes that drive tumourigenesis, thus revealing a new layer in the molecular architecture of human cancer. Recent reviews have shown that certain miRNAs are intimately involved in processes resulted in NPC^7^. However, there are only a few publications about miRNA profiles in NPC^8–10^. Chen *et al*.^8^ reported that 35 miRNAs, as determined by real-time PCR, were found to be significantly altered in 13 snap-frozen NPC tissues after surgical resection against 9 adjacent normal tissues from the same patients^8^. Several known oncogenic miRNAs, including miR-17–92 cluster and miR-155, are among the miRNAs up-regulated in NPC. Tumour suppressive miRNAs, including miR-34 family, miR-143, and miR-145, are significantly down-regulated in NPC. Li *et al*. (2011) reported that miRNA expression profiling was performed to compare miRNA expression changes in 8 poorly differentiated squamous NPC tissues with 4 normal nasopharyngeal tissues by miRNA microarray^9^. The results found that miR-18a was overexpressed and 33 miRNAs (e.g. miR-34b, miR-34c, let-7 family) were down-regulated in NPC tissues of which these miRNAs are involved in the pathway of nervous system development and sensory perception of sound being associated with NPC development. However, the relationship between miRNAs and NPC tumourigenesis is still unclear as all published miRNA profiles shared little in common except for very few miRNAs.

MiRNA expression profiling has become an important tool to study disease pathogenesis and prognosis. In view of the importance of the NPC-related miRNAs, it is important to understand their roles in NPC tumorigenesis. In this study, we adopted real-time PCR to obtain NPC miRNA profiles from the formalin-fixed paraffin-embedded (FFPE) biopsies of undifferentiated NPC patients and normal individuals. Furthermore, the expression of selected miRNA candidate (miR-150) was also validated in the NPC patients of T2 to T4 stages and non-cancerous individuals. Finally, we further elucidate the functional role of miR-150 in NPC tumourigenesis.

## Materials and Methods

### FFPE and sera samples

FFPE samples of 24 individuals including 17 NPC patients classified as T2 to T4 cancer stage and 7 non-NPC individuals were obtained from the Pamela Youde Nethersole Eastern Hospital, Hong Kong and used for the miRNA profiling. All the tissue slides were evaluated by a pathologist, especially, the NPC patient biospy slides were affirmed with at least 70% tumour content. Besides, a total of 132 NPC patients (including 41 T2, 66 T3, and 25 T4 patients) and 28 non-cancerous human serum samples were obtained from the AoE Hong Kong NPC Research Tissue Bank in accordance with ethical approval. This study has been approved by the University and Hospital Ethics Committee. Details of the samples are listed in Tables 1 and 2.

**Table 1:** Summary of FFPE samples used in the study. (A) FFPE samples.

FFPE samples	Sex	Age	Histological classification	Pathological lymph node status	Metastasis
Patient 1	M	31	2B	3	1
Patient 2	M	27	3	1	0
Patient 3	M	40	2B	1	0
Patient 4	M	40	4B	2	0
Patient 5	M	49	3	2	0
Patient 6	M	60	4A	2	0
Patient 7	M	64	Unknown	Unknown	Unknown
Patient 8	M	71	4A	0	0
Patient 9	M	38	3	1	0
Patient 10	M	67	4A	1	0
Patient 11	M	56	2A	0	0
Patient 12	M	69	3	2	0
Patient 13	F	43	4B	3	0
Patient 14	F	65	2B	1	0
Patient 15	M	53	3	2	0
Patient 16	M	42	3	2	0
Patient 17	M	52	2A	0	0
Non-NPC control 1	Unknown	Unknown	NA	NA	NA
Non-NPC control 2	Unknown	Unknown	NA	NA	NA
Non-NPC control 3	Unknown	Unknown	NA	NA	NA
Non-NPC control 4	Unknown	Unknown	NA	NA	NA
Non-NPC control 5	Unknown	Unknown	NA	NA	NA
Non-NPC control 6	Unknown	Unknown	NA	NA	NA
Non-NPC control 7	Unknown	Unknown	NA	NA	NA

Remark:

NA: Not applicable.

**Table 2:** Summary of human sera samples used in the study.

Human sera	Number of patients
Non-cancerous controls	28
NPC patients (early stage II)	41
NPC patients (late stage III)	66
NPC patients (late stage IV)	25
Total	160

### Extraction of total RNA from FFPE tissues and RNA quality assessment

Total RNA was extracted from FFPE tissues of NPC and non-NPC patients using QIAGEN miRNeasy FFPE extraction kit according to the manufacturer’s instructions. Briefly, xylene was added to remove the paraffin from the FFPE samples. The tissues were digested with protease at 50 °C and treated with DNase. After washing, total RNA including the small miRNA fraction was eluted with distilled water. Concentration and purity of the total RNA samples were assessed using the NanoDrop ND3.0 spectrophotometer (NanoDrop Technologies Inc, Wilmington, DE) by measuring the absorbance at 260 and 280 nm. RNA integrity was assessed by the small RNA assay kit (Agilent Technologies) and Agilent 2100 Bioanalyzer (Agilent Technologies, Palo Alto, CA).

### MicroRNA profiling of NPC FFPE specimens by real-time PCR

The reverse transcription and real-time PCR analysis were performed using the Cancer MicroRNA qPCR Array with QuantiMir™ kit (SBI System Biosciences) in accordance with the manufacturers’ instructions. Briefly, total RNA (500 ng) was directly converted to cDNA with the QuantiMir™ RT System. Then, real-time PCR was performed by mixing the cDNA template with a SYBR^®^ Green Mastermix (ABI) and the SBI universal reverse primer. The SYBR^®^ Green Mastermix was aliquoted into qPCR optical plate. Then, 1 μl of individual MicroRNA-specific primers from the Primer plate was pipetted into the corresponding wells of the qPCR plate. Real-time PCR was performed in Mx3000 P™ Real-Time PCR System (Stratagene) according to qPCR cycling and data accumulation conditions as suggested by the manufacturer: 50 °C for 2 mins, 95 °C for 10 mins then 40 cycles of 95 °C for 15 sec and 60 °C for 1 min (set as data collection point). Melting curve analysis after the qPCR run was performed to assess the Tm of the PCR amplicon to verify the specificity of the amplification reaction.

### Human serum samples and RNA isolation

Total RNA was extracted from 200 μl of the NPC patient and non-cancerous human serum samples using the TRIzol^®^ LS reagent (Invitrogen) premixed with 0.5 μL of 2 μM syn-Cel-miR-39 spike-in non-human *Caenorhabditis elegans* synthetic miRNA (QIAGEN), which was used to monitor the effect of PCR inhibitors as well as serve as internal reference control. The extracted total RNA was finally resuspended in 10 μL RNase-free water. The quantity and quality of the RNA were determined at 260 and 280 nm by NanoDrop 1000 spectrophotometer (Thermo Scientific, Wilmington, DE).

### cDNA synthesis and the detection of miR-150 by real-time PCR

Reverse transcription reactions and real-time PCR analysis were performed using Exiqon universal cDNA synthesis kit (Exiqon, Woburn, MA) and SYBR^®^ Green master mix kit (Exiqon), respectively, in accordance with the manufacturer’s instructions. The cDNA was firstly diluted with RNase-free water at the ratio of 1:40 and then determined by real-time PCR in the StepOnePlus™ Real-Time PCR System (Applied Biosystems^®^). After amplification, the data was analyzed by the StepOne software v2.2.2. The miRNA abundance was measured based on comparing Ct values of samples to dilutions of a synthetic cDNA of the corresponding miRNA sequence to make a standard curve.

### Construction of synthetic miRNA standard curve for absolute miRNA quantification

The miR-150 and Cel-miR-39-3p synthetic LNA RNA oligonucleotide standards were obtained from Exiqon. Each synthetic miRNA standard solution was diluted in water to obtain 1 nM working solution for subsequent serial dilution. A primary dilution of 8.192 fM miRNA standard working solution was prepared by mixing 4 μL of 1 nM miRNA standard working solution with 118.4 μL RNAase-free water. Then, 10 serial 4-fold dilutions were made by adding 20 μL of the previous miRNA working solution to 60 μL RNase-free water. The final dilution contained only the RNase-free water was used as a no-template control. 2 μL of each synthetic miRNA standard dilutions was put up for cDNA synthesis and real-time PCR analysis as described in the above. Duplicate experiments were done for each miRNA standard. Plotting Ct values against copy number of the synthetic miRNA standard curve in log scale allows fitting of a curve that is used to estimate the miRNA copy number in the biological samples.

### Cell culture and cell transfection with anti-sense miRNA inhibitor or precursor mimics

NPC cell line HK1 was provided from the AoE HK NPC Research Tissue Bank Cell Line Repository. All cells used in this study were tested and confirmed to be mycoplasma negative; and have been authenticated using the AmpFISTR identifier PCR Amplification kit (Life Technology). HK1 cells were maintained in RPMI 1640 medium supplemented with 10% FBS and 1% PS at 37 °C humidified incubator with 5% CO_2_.

HK1 cells were transfected with Anti-miR™ miRNA Inhibitor (Anti-miR-150) or Pre-miR™ miRNA Precursor (Pre-miR-150) (50 nM) (Ambion, USA) using Lipofectamine™ 2000 in Opti-MEM^®^ I Reduced Serum Medium (Invitrogen). Total RNA and cell lysate were extracted for the indicated assays. Pre-miR™ miRNA Precursor Negative Control #1 (Pre-control) (Ambion) and anti-miR™ miRNA Inhibitor Negative Control #1 (AS-control) were used as negative controls in the experiments, respectively^11,12^.

### Cell migration assay

HK1 cells (3 × 10^4^ cells/well) with transfection of Anti-miR-150, Pre-miR-150, AS-control, or Pre-control oligonucleotide were plated onto gelatin (0.1%)-precoated 96-well plates and incubated for 20 hours. An artificial wound was created by mechanical scratching of the cell monolayer. The denuded area in each well was captured (t = 0 hr) using Motic Image Plus 2.0 software (Motic Instruments Inc., Canada). Cells were then incubated for further 20 hours and the denuded area of each well was captured again. Images at 0 and 20 hours were analyzed using Image J software. The cell motility was expressed as percentage of recovery.

$$ \% \,{\rm{of}}\,{\rm{recovery}}=[({{\rm{A}}}_{{\rm{t}}=0}\,-\,{{\rm{A}}}_{{\rm{t}}=20})/{{\rm{A}}}_{{\rm{t}}=0}]\times 100 \% $$

where, A_t=0_ is the area of wound measured immediately after scratching, A _t=20_ is the area of wound measured 20 hours after scratching. Each sample was assayed in quadruplicates and the assay was repeated in triplicates^13,14^.

### Cell invasion assay

The invasive potential of Anti-miR-150 or Pre-miR-150-transfected HK-1 cells was studied using Transwell chamber with 6.5 mm diameter polycarbonate filter (8 mm pore size, NUNC). Briefly, the upper and lower surfaces of the filter were coated with GFR-Matrigel (diluted in PBS, 1:30 and 1:100, v/v), respectively. Transfected cells (5 × 10^4^ cells/well) were loaded into the upper wells with culture medium containing 1% FBS. The transwell chambers were sequentially inserted into 24-well plates containing culture medium with 10% FBS and incubated at 37 °C for 5 h. The invaded cells were fixed with methanol and stained with crystal violet; those on the top side of the membrane were wiped off by cotton swab. The invaded cells were captured using stereomicroscope (Olympus SZX16) with digital camera (Olympus DP17) (Olympus America Inc.), quantified by dissolving the cells on the membranes in 500 μl 10% acetic acid, and measuring the OD values at 570 nm by microplate reader (Infinite F200, Tecan, Mannedorf Switzerland). Each sample was assayed in triplicates and the assay was repeated in triplicates^15^.

### Western blot analysis

Equal amount of protein samples (20 μg) extracted from the cells were separated by SDS-PAGE and transferred onto a nitrocellulose membrane. After blotting, the membrane was probed with primary antibodies (1:1000) against E-cadherin (Upstate Biotechnology, USA) and fibronectin and vimentin (Sigma), and subsequently incubated with secondary antibody (1:2000). The membrane was then washed and antibodies against E-cadherin (Upstate Biotechnology, USA) and fibronectin and vimentin (Sigma), and subsequently incubated with secondary antibody. The membrane was then washed and visualized by ECL detection system (Bio-Rad Laboratories). Actin expression was used as protein loading control. Densitometry quantification was performed in Region of Interest (ROI) approach using Kodak Digital Science 1D, v.3.0.0. (Scientific Imaging Systems, USA). Three independent experiments were carried out to study the protein expression.

### Data and statistical analysis

For the analysis of FFPE profiling data, the fold change was calculated using 2^−ΔΔCT^ method in accordance with the manufacturer’s instructions. In general, cycle thresholds were set within the exponential phase of the amplification plots with software automatic baseline settings. As recommended by the manufacturer, the relative expression ratio of miRNAs ≥3-fold was considered to be up-regulated while the relative expression ratio of miRNAs ≤0.33 fold (equivalent to −3 fold) was considered to be down-regulated. The data was further analyzed with DataAssist™ v3.01 (Applied Biosystems) to generate the volcano plot. The incidence % was defined as the percentage of number of NPC patients with miRNA deregulation to the total number of patients under the study. The expressions of the miRNAs in NPC tissues and non-NPC tissues were compared using paired student’s *t*-test. Data are presented as mean ± standard deviation (SD). A *p*-value less than 0.05 was considered statistically significant. For the analysis of human serum data, all real-time PCR analysis of the miRNA standard curve was representative of duplicate independent experiments. Data were presented as mean ± standard derivation of four data points from duplicate independent experiments. Statistical analysis was performed with Prism v5.03 and the differences were considered statistically significant, when the *p*-value was less than 0.05 by using the Student’s *t*-test method.

## Results

### FFPE tissue miRNA profiling

In this study, miRNAs have been successfully extracted from all FFPE specimens for subsequent real-time PCR analysis. To identify miRNAs differentially expressed in NPC, we analyzed the expression levels of 95 human cancer related-miRNAs in 17 NPC tissues from T2 to T4 stage cancer and 7 non-NPC tissues by real-time PCR, which has been proven to offer high sensitivity and specificity for the quantification of mature miRNAs.

**Figure 1:**
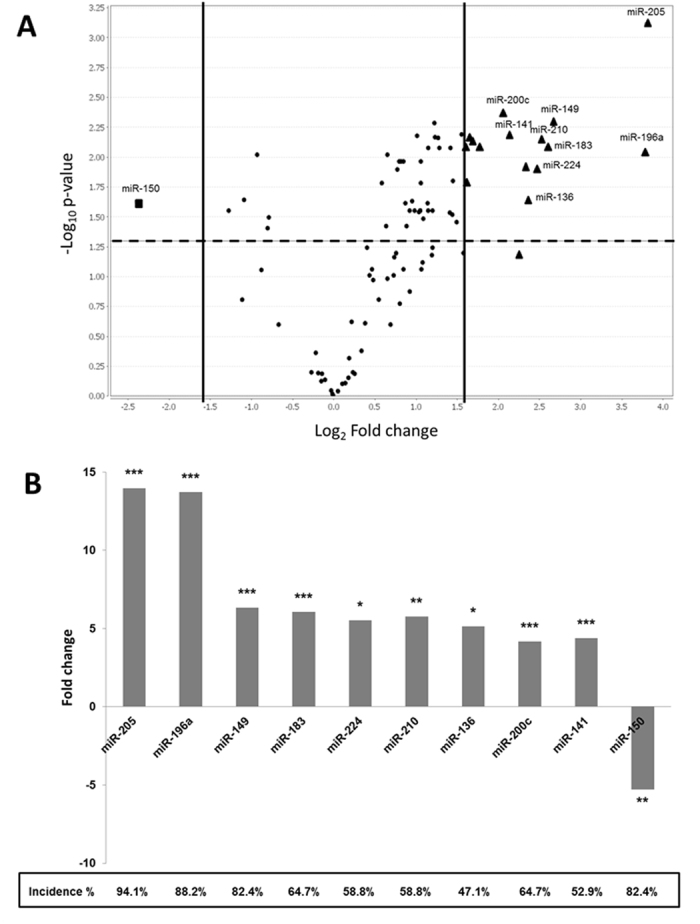
miRNA profiling of NPC and non-NPC FFPE samples by real time PCR. (**A**) The fold changes of miRNA expressions were calculated and presented as a volcano plot. The two vertical solid lines and the horizontal dotted line indicate the significance cut-offs of three-fold differential expression and *p*-value of 0.05, respectively. A total of 16 miRNAs were found to be significantly up-regulated (▲), while only 1 miRNA was down-regulated (■). (**B**) By considering the percentage of incidence, which was defined as the percentage of number of NPC patients with miRNA deregulation to the total number of patients under the study, 10 differentially expressed miRNAs (miRNAs labelled in (**A**)) out of 95 cancer related miRNAs were confirmed to be significantly modulated. Nine miRNAs were over-expressed (fold-change ≥3); while only one miRNA was under expressed (fold-change ≤3) in NPC patients.

After normalization to the control U6 snRNA expression, the differential expression of 95 miRNAs between NPC and non-NPC tissues was compared. As shown in Fig. 1A, 17 miRNAs were found to be differentially expressed among the NPC specimens. Among them, 16 miRNAs were significantly over-expressed with fold changes more than 3-fold, while one miRNA was down-regulated with a fold change of less than 3-fold and the statistical significance was evaluated by pair *t*-test. The incidence of miRNA deregulation between non-NPC controls and NPC patients group was also calculated in Fig. 1B. The incidence of up and down regulation of these ten miRNAs, including miR-205 (94.1%), miR-196a (88.2%), miR-149 (82.4%), miR-183 (64.7%), miR-224 (58.8%), miR-210 (58.8%), miR-136 (47.1%), miR-200c (64.7%), miR-141 (52.9%) and miR-150 (82.4%) between non-NPC controls and NPC patients ranges from about 47% to 94% incidence rate. By considering the three-fold changes and the 50% incidence rate as the cut-off values, ten candidate miRNAs were identified. They were miR-205, miR-196a, miR-149, miR-183, miR-224, miR-210, miR-136, miR-200c, miR-141 and miR-150. The biological significance of these miRNAs with similar deregulation as NPC is summarized in Table 3.

**Table 3:** Summary of the biological function of miRNA candidates.

miRNAs	Deregulation in Cancer	Functional Significances	References
miR-205	↑	modulates cellular invasion and metastasis and EMT in ESCC, bladder and prostate cancer	Chen *et al*.^8^ Matsushima *et al*.^32^ Tran *et al*.^33^ Massoner *et al*.^34^
miR-196a	↑	promotes cell survival, metastasis and oncogenic phenotype in colorectal and gastric cancer ESCC	Schimanski *et al*.^35^ Tsai *et al*.^36^ Wang *et al*.^37^
miR-149	↑	promotes EMT and invasion in NPC cell lines	Luo *et al*.^18^
miR-183	↑	modulates the invasive and metastatic propensities of lung adenocarcinoma, promotes tumour cell dissemination, and tumour initiating capacity of pancreatic and colorectal cancer cells, and migration of cancer stem cells	Yang *et al*.^38^ Wellner *et al*.^39^
miR-224	↑	promotes cell proliferation, migration, invasion and anti-apoptosis in HCC	Zhang *et al*.^40^
miR-210	↑	modulates the cell survival and angiogenesis which facilitate the metastasis, induce the metastatic potential of HCC	Tsurumi *et al*.^41^ von Deetzen *et al*.^42^ Ellermeier *et al*.^43^
miR-136	↑	regulates glioblastoma cell growth and migration, promotes cell growth in NSCLC	Jeansonne *et al*.^44^ Shen *et al*.^45^
miR-200c & miR-141	↑	promotes migration and invasion ability of NPC, HNSCC, and NSCLC, promotes EMT in colorectal cancer, promotes angiogenesis in NSCLC	Burk *et al*.^46^ Vrba *et al*.^47^ Hur *et al*.^48^ Tamagawa *et al*.^49^ Tejero *et al*.^50^ Zhang *et al*.^22^
miR-150	↓	induces EMT and contributes to malignant potential in epithelial ovarian and colorectal cancer	Pizzini *et al*.^51^ Jin *et al*.^52^ Yokobori *et al*.^27^

Remark: “**↑**” indicates up-regulation; “**↓**” indicates down-regulation.

Head and Neck Squamous Cell Carcinoma (HNSCC).

Non-small Cell Lung Cancer Adenocarcinoma (NSCLC).

Esophageal Squamous Cell Carcinoma (ESCC).

Hepatocarcinoma Cells (HCC).

### miR-150 expression in human sera

**Figure 2:**
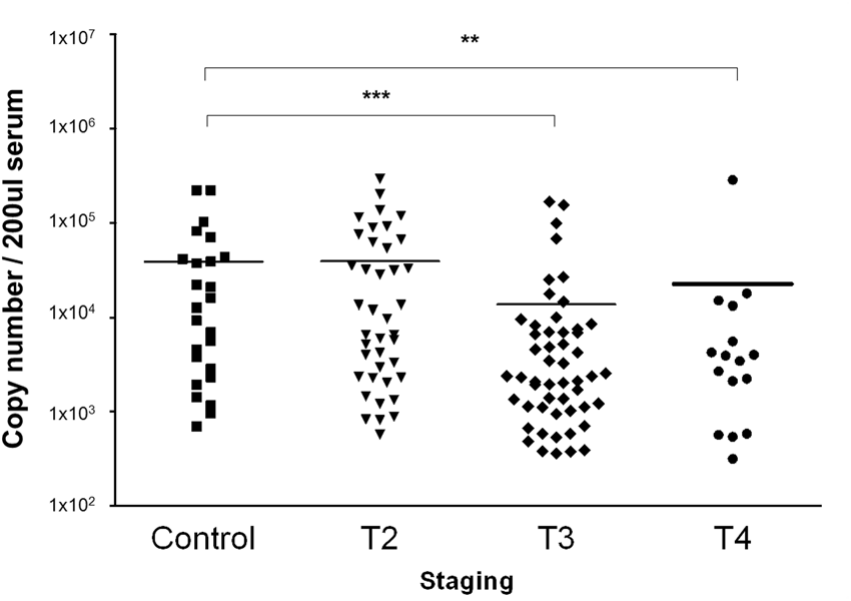
Scatter dot plot of the expression level of miR-150 in the human sera of NPC patients and non-cancerous controls. The line indicated the mean expression values. **p < 0.01, *p < 0.05.

The expression of miR-150 in 132 NPC patients of different cancer stages and 28 non-cancerous controls was evaluated by the real-time PCR. It was found that miR-150 expression level in the serum samples from patients with T3 and T4 tumours were significantly lower than those from T2 patients and non-cancerous controls (Fig. 2). There was about 3-fold decrease of miR-150 expression level by comparing T2 with T3 (*p* < 0.01). The incidence rate of miR-150 in non-cancerous control was up to 82%. This result is concordant with the FFPE profiling data. The decreasing trend of miR-150 expression level in the sera of T2-T4 patients is consistent with that in the FFPE patients of T2-T4 stages, suggested that there would be a strong correlation of the change of miR-150 expression between tissue and sera.

### Functional characterization of miR-150 in HK1 cells

**Figure 3:**
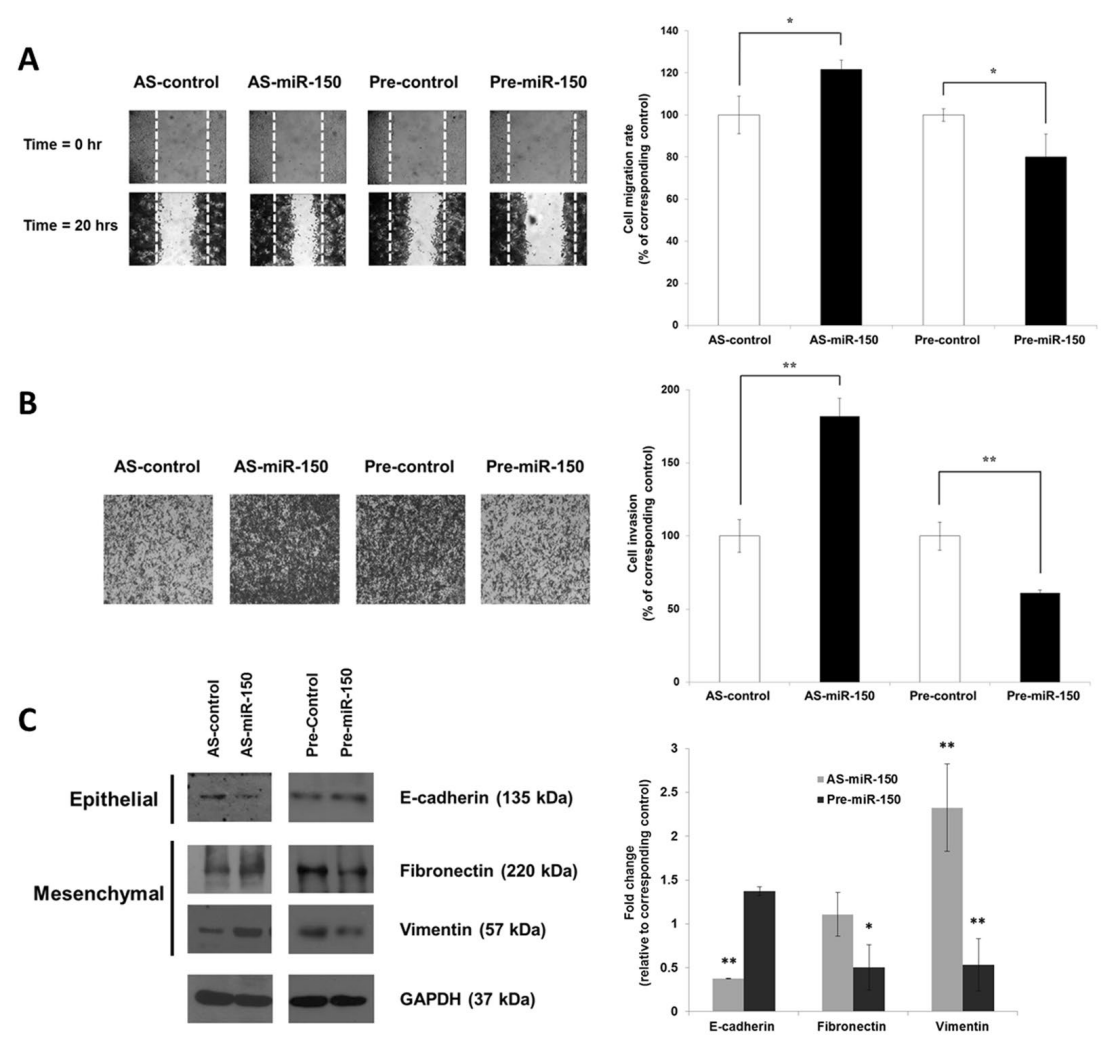
miR-150 regulate *in vitro* cell migration and invasion of HK-1 cells and EMT protein expression. (**A**) Cell migration assay. Cells transfected with various oligonucleotides (50 nM) including AS-control, Anti-miR-150, Pre-control or Pre-miR-150 were seeded and the cell monolayer was denuded by mechanical scratching. The denuded areas were captured at 0 and 20 hours after scratching (left) and the cell motility was calculated (right). (**B**) Cell invasion assay. MicroRNA oligonucleotides transfected cells were added to the transwell and invaded cells were stained (left) and quantitated by colorimetric measurement (right). Values were presented as mean ± SD. At least three independent experiments were carried out, and each sample was performed in triplicate. (**C**) Western blot analysis. Total cell lysates were harvested from transfected cells and epithelial markers – E-cadherin and mesenchymal marker – vimentin and fibronectin expressions was determined (left) and densitometric quantitated (right). *p < 0.05; **p < 0.01 compared with corresponding control.

To understand the functional role of miR-150 in NPC motility, cell migration and invasion assays were conducted. As shown in Fig. 3A, when the HK1 cells were overexpressed with miR-150 by transfecting with Pre-miR-150, their motility was suppressed about 17%, when comparing with the Pre-control group. By contrast, the cell motility was raised about 16% after transfecting with miR-150 inhibitor. This suggested that miR-150 acted as a repressor in the nasopharyngeal cell motility. A similar result was observed in the invasion assay, in which the AS-miR-150 transfected cells presented a higher invasive potential; there was about 36% increment when comparing with the control group (AS-control). The overexpression of miR-150 cells also resulted in the significant reduction (about 23%) of cell invasion (Fig. 3B).

As the expression of epithelial markers (e.g. E-cadherin) and mesenchymal markers (e.g. fibronectin, N-cadherin, vimentin) are important indicators of cancer metastasis, Western blotting was employed to detect the EMT marker proteins expressions. The data showed that the expression of E-cadherin was increased, but the expression of fibronectin and vimentin were decreased in the miR-150 overexpressing HK1 cells (Fig. 3C). In contrast, knockdown of miR-150 resulted in the decrease of E-cadherin, but increased the expression of fibronectin and vimentin in HK1 cells when comparing with AS-control. These data suggested that the miR-150 possibly acts as a molecular mediator in NPC pathogenesis by modulating the EMT status of NPC cells.

## Discussion

Archived FFPE specimens represent an excellent resource for biomarker discovery because documented clinico-pathological histories such as clinical symptoms, treatment outcomes and the long-term follow-up are available. With an objective of exploring the NPC-related miRNAs, a profiling analysis of topical cancer miRNAs on NPC FFPE tissues was conducted. We used a miRNA real-time PCR platform that covered 95 human cancer related miRNAs to investigate the expression profiles of miRNA in 17 NPC patients with undifferentiated tumours and 7 non-NPC patients. By considering the fold-change threshold and percentage of incidence, among the 95 screened miRNAs which have functional significance with regard to potential roles in cancer, cell development and apoptosis, ten miRNAs (miR-205, miR-196a, miR-149, miR-183, miR-224, miR-210, miR-150, miR-136, miR-200c and miR-141) were identified which may play a putative role in cancer development, metastasis and have potential as biomarkers for the detection of NPC.

In this study, we tried to compare our expression profile with that of other cancer types. Promisingly, a unique miRNA signature of NPC has been obtained, when comparing against head and neck squamous carcinoma (HNSCC), oesophageal squamous cell carcinoma (ESCC), lung cancer, breast cancer and pancreatic cancer (Table 4). Even though, the NPC is originated from nasopharynx and commonly spread to lymph nodes in neck nearby region, the pathology, aetiology and therapeutic response are different from other HNSCC and ESCC. As shown in Table 4, the expression pattern or individual expression of miRNAs candidates in NPC is very different with HNSCC and ESCC. Among these 10 miRNAs candidates, 6 (miR-196a, miR-183, miR-224, miR-136, miR-200c and miR-150) and 3 (miR-136, mi141 and 150) miRNAs have no data reported in HNSCC and ESCC, respectively. Moreover, expression pattern of miR-205 and miR-149 in HNSCC and ESCC; and miR-210 in ESCC, were opposite to our data. Our miRNA profile further indicated that the underlying genetic aberrations and carcinogenesis pathways of NPC are very different from these similar cancer cases^16^. Besides, we also compared our data with other NPC profiling studies^8–10^, but data was non-concordant with their results. As mentioned by Li *et al*., the reasons of discrepancies on the findings would be numerous. As shown in Table 5

**Table 4:** Comparing the miRNA expression signature of NPC with other cancer types.

**miRNAs**	**NPC FFPE tissue (in this study)**	**Head and Neck Squamous Carcinoma (HNSCC)**	**Esophageal Squamous Cell Carcinoma (ESCC)**	**Lung cancer**	**Breast cancer**	**Pancreatic cancer**
205	↑	↓	↓	↑	↓	↑
196a	↑	ND	↑	↑	↑	↑
149	↑	↓	↓	↑	↑	—
183	↑	ND	↑	↓		—
224	↑	ND	—	↓	ND	↑
210	↑	↑	↓	↑	↑	↑
136	↑	ND	ND	↑	↑	↓
200c	↑	ND	↑	ND	↓	—
141	↑	↑	ND	↑	↓	—
150	↓	ND	ND	↑	ND	↑

Remark: “↑”: Upregulation. “↓”: Down regulation. “—”: No significant difference between disease tissue and normal tissue. “ND”: No data reported.

, the clinical samples used by each group were different in nature and processing procedures such as FFPE and snap frozen tissues, and sources of origin. Secondly, the detection methods including microarray or real-time PCR and data processing procedures (threshold cut-off) were different. Thirdly, the choice of normal reference was also different. We believe these heterogeneities greatly contributed to the discrepancy in the data comparison. Moreover, we know that the clinical outcomes of the treatment in differentiated and undifferentiated type NPC are very different. The former is insensitive to radiotherapy and more invasive, while the latter is very sensitive to radiotherapy. This may be due to the difference of genetic modulation of tumourigenesis of these two types of NPC. Obviously, our miRNA profile showed consistency to this hypothesis with significant difference from the differentiated NPC miRNA profile. In some circumstances, it represented the difference in genetic aberrations and carcinogenesis pathways among them.

**Table 5:** Comparing the miRNA profiling data with other NPC publications.

**miRNAs**	**Dataset from this study**	**Dataset from Li (Li et al., 2011)**	**Dataset from Chen (Chen et al., 2009)**	**Dataset from Sengupta (Sengupta et al., 2008)**
**205**	**↑** (13.0)	**X**	**↑** (4.0)	**—** (0.99)
**196a**	**↑** (12.9)	**X**	**ND**	**↓** (0.62)
**149**	**↑** (6.8)	**X**	**ND**	**—** (0.95)
**183**	**↑** (6.0)	**X**	**ND**	**↑** (2.06)
**224**	**↑** (5.7)	**X**	**ND**	**↑** (4.1)
**210**	**↑** (5.0)	**↓** (−14.27)	**ND**	**—** (1.0)
**136**	**↑** (4.8)	**X**	**ND**	**↑** (1.7)
**200c**	**↑** (4.1)	**X**	**ND**	**—** (1.0)
**141**	**↑** (3.5)	**X**	**ND**	**—** (1.2)
**150**	**↓** (−5.3)	**↓** (−4.47)	**ND**	**↑** (1.52)
**Study groups**	17 NPC /7 biopsy-negative FFPE tissues	8 NPC/ 4 normal donors snap-frozen NP tissues	13 NPC snap-frozen tissues / 9 paired adjacent normal NP tissues from patients	31 NPC samples/ 10 normal NP tissues (from 2 normal donors, 4 biopsy-negative tissues, 4 paired adjacent normal NP tissues)
**NPC Staging**	II, III, IV	II, III, IV	Unknown	II
**Differentiation**	Undifferentiated	Poorly differentiation	Unknown	Unknown
**Origin**	Hong Kong	Guangxi province of China	Taiwan	Taiwan
**Test method**	Whole tissue/ Real time PCR	Whole tissue/ miRNA microarray	Whole tissue/ Real time PCR	Laser-capture microdissection /miRNA microarray
**Deregulation cut-off fold change**	± 3 folds	q-value ≤ 0.05	± 3 folds	± 1.5 folds

Remark: “**↑**”: Up regulation. “**↓**”: Down regulation. “**—**”: No significant difference. “X”: Either not expressed or non-detectable. “ND”: No data reported.

In our miRNA profile, a common pattern was recognized that the 10 miRNA candidates are closely related to the tumourigenicity and metastasis. They have been found to promote tumour metastasis through the direct promotion of invasion, migration and epithelial-mesenchymal-transition (EMT); or indirect stimulation via induction of angiogenesis and cell survival, in different cancers such as ESCC, NSCLC, HNSCC, NPC, prostate, bladder, gastric and colorectal cancer (Table 3). Among them, miR-141, 149, 200c, and 205 have been reported in the NPC models. Qu *et al*. demonstrated that introducing miR-205 into the parental cell line CNE-2 can suppress phosphatase and tensin homolog (PTEN) protein expression, followed by activation of AKT, increased number of foci formation and reduction of cell apoptosis after irradiation. Whereas knocking down miR-205, the inhibition of PTEN expression in the radio-resistant NPC cell line CNE-2R reconciled the inhibition of PTEN expression and the increase of cell apoptosis^17^. Our results showed that miR-205 is highly expressed in NPC patients who have been clinically confirmed not to be metastatic. Further investigation of the relative expression difference of miR-205 between metastatic and non-metastatic NPC patients may be promising as a mean of diagnosing metastatic disease as well as accessing the outcome of radiotherapy. For the miR-149, it has been shown to overexpress in NPC cells, 5–8F and 6–10B, as compared to normal immortalized nasopharyngeal epithelial NP69. Its over-expression not only increases cell proliferation but also promotes EMT and invasion^18^. The study proved that miR-149 may be involved in NPC metastasis through regulation of EMT, since the up-regulation of miR-149 caused the reduction of E-cadherin expression which is known to associate with cell mobility. The miR-141 and miR-200c, which belong to the miR-200 family, have been shown to play a critical role in the EMT in various malignancies including breast, renal clear cell, gastric, and bladder^19^. A recent publication demonstrated that ZFHX1B (also known as SIP1 and ZEB2), a transcriptional repressor for CDH1/E-cadherin, is a target of both miR-141 and -200c. Over-expression of miR-141 and miR-200c caused down-regulation of ZFHX1B and up-regulation of E-cadherin in two renal carcinoma cell lines^20^. E-cadherin, a tumour suppressor gene, is important for the formation and maintenance cell-cell adhesion in epithelial tissues through calcium-dependent interaction. Loss of E-cadherin-mediated-adhesion is associated with tumour initiation, progression and the transition from benign lesions to invasive, metastatic cancer^21^. Zhang *et al*. reported that miR-141, found to be up-regulated in NPC specimens in comparison with normal nasopharyngeal epithelium, is involved in NPC-related genes network by targeting BRD3, PTEN and UBAP1. Inhibition of miR-141 could affect cell cycle, apoptosis, cell growth, migration and invasion in NPC cells^22^. Studies showed that lung and nasal epithelium clone 1 (SPLUNC1) gene, a tissue-specific gene of nasopharyngeal epithelia, is down-regulated in NPC^22–24^. SPLUNC1 protein, expressed in the serous glands and epithelium of the upper respiratory tract, is an innate immunity-defensive secretary protein which binds to bacterial lipopolysaccharide to inhibit *Pseudomonas aeruginosa* and Epstein-Barr virus^25^. It was confirmed that miR-141 and miR-205 were dramatically down-regulated in Splunc 1 expressed cell. On the other hand, inhibition of miR-141 significantly decreases the viability of Splunc 1-expressed cells with cell cycle arrest in the G0–G1 phase and decreases in cell migration and invasion ability. These suggested that miR-141 might play an important role as an oncogene in NPC tumourigenesis^22^. From our data, we found that both miR-200c and miR-141 were over-expressed in over half of the NPC patient group, which was clinically diagnosed as metastasis stage 0. Monitoring the change of their expression levels may serve as an indicator for the disease progression as it has been proven that high level of both miR-141 and 200c will lead to increased expression of E-cadherin, eventually causing inhibition on tumour progression and metastasis.

Apart from the up-regulated miRNAs, there was only one miRNA (miR-150) that showed under expression and was being detected in the profiling study. Importantly, its expression was validated in NPC patient serum samples (Fig. 2). It has been reported that miR-150 may play a role in the late stage of pathogenesis through facilitating metastasis and also associating with poor overall survival. A recent publication demonstrated that miR-150 functioned as tumour suppressor in a mouse xenograft model and lower expression of miR-150 was found in ESCC than in normal esophageal mucosa^26^. The study proved that down-regulation of miR-150 contributed to malignant potential in ESCC through targeting EMT inducer, ZEB1 and was also associated with poor prognosis and tumour progression^27^. However, deregulation of miR-150 in NPC was still uncertain. In this study, we were interested to know the functional role of miR-150 in NPC tumourigenesis. Through a series of *in vitro* gain-of-function and loss-off-function assays, diminished miR-150 was found to increase the HK-1 cell motility and invasiveness; while overexpression of miR-150 could significantly reserve the cellular behavior (Fig. 3A and B). Western blotting analysis indicated that EMT marker proteins including E-cadherin, fibronectin and vimentin could be involved. Concomitant result showed that the suppression of E-cadherin protein and increment of fibronectin and vimentin proteins were found in the miR-150 knockdown HK-1 cells; while increase of E-cadherin and decrease of fibronectin and vimentin protein expression were detected in the miR-150 overexpressed HK-1 cells. Obviously, dysregulation of miR-150 increased NPC cells motility through modulating EMT proteins expression. In fact, the EMT, where malignant tumour cells switch from a polarized epithelial phenotype to a highly motile mesenchymal phenotype, is one of the paramount steps during tumour progression^28,29^. We believed that miR-150 would be a key molecular regulator in NPC tumourigenesis.

## Conclusion

By using a miRNA profiling technique, a series of remarkable miRNAs related to NPC have been found with high incidence percentage in the NPC patient group against non-NPC controls in this current study. Finding out a cancer-specific miRNA usually is difficult as each miRNA has multiple target genes and can function as tumour suppressor or oncogene in different cancers. Some of the researchers suggested the approach of monitoring the change of relative expression level of certain miRNAs to predict the prognosis of cancer^30,31^. For example, monitoring of the down-regulation of miR-133a, miR-133b, miR-205 and let-7d in HNSCC tumours served as indicators in the prognosis of HNSCC patients. Our data strengthened the feasibility of screening targeted miRNAs as a non-invasive diagnostic and first line screen in the case of NPC. Furthermore, the gain-of-function and loss-of-function *in vitro* studies clear demonstrated the importance of miR-150 in NPC cell migration, invasion, and EMT, which are classified as key steps in tumour metastasis. Through identifying the functional role of targeted miRNA, it also provided a foundation for further understanding of disease progression and therapeutic targets.
